# Bacterial supergroup‐specific “cost” of *Wolbachia* infections in *Nasonia vitripennis*

**DOI:** 10.1002/ece3.9219

**Published:** 2022-09-13

**Authors:** Alok Tiwary, Rahul Babu, Ruchira Sen, Rhitoban Raychoudhury

**Affiliations:** ^1^ Department of Biological Sciences Indian Institute of Science Education and Research, Mohali (IISER Mohali) Punjab India; ^2^ Zoological Survey of India Kolkata West Bengal India; ^3^ Sri Guru Gobind Singh College Chandigarh India

**Keywords:** cytoplasmic incompatibility, life‐history traits, *Nasonia*, prospermatogeny, quantitative PCR, *Wolbachia*

## Abstract

The maternally inherited endosymbiont, *Wolbachia*, is known to alter the reproductive biology of its arthropod hosts for its own benefit and can induce both positive and negative fitness effects in many hosts. Here, we describe the effects of the maintenance of two distinct *Wolbachia* infections, one each from supergroups A and B, on the parasitoid host *Nasonia vitripennis*. We compare the effect of *Wolbachia* infections on various traits between the uninfected, single A‐infected, single B‐infected, and double‐infected lines with their cured versions. Contrary to some previous reports, our results suggest that there is a significant cost associated with the maintenance of *Wolbachia* infections where traits such as family size, fecundity, longevity, and rates of male copulation are compromised in *Wolbachia*‐infected lines. The double *Wolbachia* infection has the most detrimental impact on the host as compared to single infections. Moreover, there is a supergroup‐specific negative impact on these wasps as the supergroup B infection elicits the most pronounced negative effects. These negative effects can be attributed to a higher *Wolbachia* titer seen in the double and the single supergroup B infection lines when compared to supergroup A. Our findings raise important questions on the mechanism of survival and maintenance of these reproductive parasites in arthropod hosts.

## Graphical abstract

*Wolbachia* infected males show a reduction in the number of copulation and quicker sperm depletion.

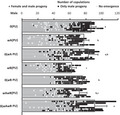

## 1 INTRODUCTION

*Wolbachia* are maternally inherited, obligatory intracellular, endosymbionts of the order Rickettsiales (Hertig & Wolbach, 1924), which are widely found in arthropods and filarial nematodes (Bandi et al., 1998; Rousset et al., 1992; Weinert et al., 2015). To enhance their own transmission, these bacteria often alter host reproductive biology with mechanisms like male‐killing, feminization, parthenogenesis, and cytoplasmic incompatibility (CI) (Werren et al., 2008). While CI leads to an increase in the number of infected individuals in the population, male‐killing, and feminization shifts the offspring sex ratio towards females, which is the transmitting sex for *Wolbachia*. Thus, *Wolbachia* increases the fitness of the infected hosts, over the uninfected ones, as it increases its own rate of transmission. The vast majority of *Wolbachia*‐host association studies reveal many negative effects on the hosts. In addition to reproductive traits, many other life‐history traits like longevity and developmental time are also known to be compromised. A review of such negative effects of *Wolbachia* on hosts where CI is prevalent is presented in Table 1. In *Trichogramma kaykai* and *T. deion*, the infected (thelytokous) line shows reduced fecundity and adult emergence rates than the antibiotically cured (arrhenotokous) lines (Hohmann et al., 2001; Tagami et al., 2001). *Leptopilina heterotoma*, a *Drosophila* parasitoid, has adult survival rates, fecundity, and locomotor performance, of both sexes, severely compromised in *Wolbachia*‐infected lines (Fleury et al., 2000). Larval mortality has been observed in both sexes of insecticide‐resistant *Wolbachia*‐infected lines of *Culex pipiens* (Duron et al., 2006). *Wolbachia* infections can also result in a range of behavioral changes and altered phenotypes in *Aedes aegypti* (Turley et al., 2009). While these cases highlight a parasitic effect of *Wolbachia*, there are several examples where no such effect is discernible (Hoffmann et al., 1996). Moreover, there are also examples where *Wolbachia* has now become a mutualist and offers specific and quantifiable benefits to its host. One such example of an obligate mutualism with *Wolbachia* has been reported in the common bedbug *Cimex lectularius* where *Wolbachia*, found to be localized in bacteriomes, provides essential B vitamins needed for growth and fertility (Hosokawa et al., 2010). Such examples of arthropod‐*Wolbachia* mutualism have now been reported from various arthropod taxa (Miller et al., 2010; Pike & Kingcombe, 2009). This shift from parasitic to mutualistic effect can also happen in facultative associations as seen in *Drosophila simulans*, where within a span of just two decades, *Wolbachia* has evolved from a parasite to a mutualist (Weeks et al., 2007).

**TABLE 1:** Negative fitness effects of CI‐inducing *Wolbachia*

*Wolbachia* supergroup	Genera	Species	*Wolbachia* strain	Host sex	Negative effect	Reference
A	*Drosophila*	*D. melanogaster*	A*‐w*MelPop	Female/Male	Tissue degeneration, reduced life span	Min and Benzer (1997), Reynolds et al. (2003)
			A*‐w*MelPop	Female/Male	Decreased response to food cues	Peng et al. (2008)
			A‐*w*Mel	Female	Reduced body size	Hoffmann et al. (1998)
			A*‐w*Mel	Female	Reduced fecundity after a dormancy period	Kriesner et al. (2016)
		*D simulans*	A*‐w*Ri	Female	Reduction in fecundity	Hoffmann and Turelli (1988), Hoffmann et al. (1990)
			A*‐w*Ri	Female	Reduction in fecundity	Snook et al. (2000)
			A*‐w*Ri	Male	Lesser sperm cysts, reduced fertility	Snook et al. (2000)
			A‐*w*Ha	Female	Reduction in fecundity	Fytrou et al. (2006)
			A‐*w*Ha	Female/Male	Reduced thorax length, reduction in an immune response against parasitoid infection	Fytrou et al. (2006)
			A*‐w*Ri	Male	Reduced sperm competition	De Crespigny and Wedell (2006)
		*D. suzukii*	A‐*w*Suz	Female	Reduced progeny family size	Hamm et al. (2014)
B	*Aedes*	*A. albopictus*	A‐*w*Alb, B‐*w*Alb	Female	Reduced life span, reduction in fecundity	Islam and Dobson (2006), Suh et al. (2009)
		*A. aegypti*	A‐*w*MelPop	Female	Reduced life span, reduction in fecundity	Ross et al. (2019)
			A‐*w*MelPop	Female/Male	Reduced life span	McMeniman et al. (2009)
			A*‐w*MelPop	Female	Reduction in fecundity, reduced blood‐feeding success	Allman et al. (2020), Turley et al. (2009)
	*Culex*	*C. pipiens*	B‐*w*Pip	Female/Male	Embryonic mortality	Duron et al. (2006)
		*C. quinquefasciatus*	B‐*w*Pip	Female	Reduced fecundity	Almeida et al. (2011)

The negative effects of *Wolbachia* on their hosts are not unexpected. The presence of bacteria within a host entails sharing of nutritional and other physiological resources (Kobayashi & Crouch, 2009; Whittle et al., 2021), especially with *Wolbachia*, as they are obligate endosymbionts and cannot survive without cellular resources derived from their hosts (Foster et al., 2005; Slatko et al., 2010). Accordingly, *Wolbachia* is known to compete with the host for key resources like cholesterol and amino acids in *A. aegypti* (Caragata et al., 2014). The precise molecular mechanisms of many of these negative effects have not been ascertained and are generally ascribed to partitioning‐off of host nutrients for its benefit, but what is clear is that *Wolbachia* infections can impose severe nutritional demands on their hosts (Ponton et al., 2014). However, it is also known that *Wolbachia* can elicit antipathogenic responses from their hosts where the host resistance or tolerance to the infection increases (Zug & Hammerstein, 2015). For example, *Wolbachia* induces host methyltransferase gene *Mt2* towards antiviral resistance against Sindbis virus in *D. melanogaster* (Bhattacharya et al., 2017). *Wolbachia* can utilize the immune deficiency (IMD) and Toll pathways (Pan et al., 2018) and increase reactive oxygen species (ROS) levels in *Wolbachia*‐transfected *A. aegypti* mosquitoes, inhibiting the proliferation of the dengue virus (Pan et al., 2012). Such immune responses require additional allocation of resources, which can further affect other physiological traits of the host. This concept of a “cost of immunity” is well‐established and suggests a trade‐off between immunity and other life‐history traits (Zuk & Stoehr, 2002). For example, elevated ROS levels negatively affect many host traits like longevity and fecundity (Dowling & Simmons, 2009; Monaghan et al., 2009; Moné et al., 2014; Selman et al., 2012). Thus, there is sufficient evidence to conclude that *Wolbachia* can have substantial negative effects on the overall fitness of its host.

One of the arthropod hosts infected by *Wolbachia* is the parasitoid wasp *Nasonia vitripennis*. *N. vitripennis*, being cosmopolitan, has been used to study *Wolbachia* distribution, acquisition, spread, and *Wolbachia*‐induced reproductive manipulations (Landmann, 2019; Werren et al., 2008). However, the effect of the endosymbiont on the life‐history traits of this wasp remains poorly understood with conflicting reports. *N. vitripennis* harbor two *Wolbachia* supergroup infections, one each from supergroup A and supergroup B (Perrot‐Minnot et al., 1996), and the presence of these two infections has been found in all lines of *N. vitripennis* from continental North America to Europe (Raychoudhury et al., 2010), indicating that it has reached fixation across the distribution of its host. The two *Wolbachia* in *N. vitripennis* together cause complete CI, but single infections of supergroup A *Wolbachia* cause incomplete CI while supergroup B infections still show complete CI (Perrot‐Minnot et al., 1996). In some *N. vitripennis* lines, *Wolbachia* has been reported to cause enhanced fecundity (Stolk & Stouthamer, 1996), but a similar effect has not been observed in some other lines (Bordenstein & Werren, 2000). In this study, we investigate, what, if any, are the negative effects of CI‐inducing *Wolbachia* infections in *N. vitripennis*. We investigate the effects of *Wolbachia* infections in a recently acquired line of *N. vitripennis* from the field. This line, like other *N. vitripennis* lines, has two *Wolbachia* infections, one each from the supergroup A and B. Sequencing of the five alleles from the well‐established multi‐locus strain typing (MLST) system (Baldo et al., 2006) reveals no sequence variation with other *Wolbachia* strains done previously (Prazapati, personal communication) indicating, that this new *N. vitripennis* line is also infected by the same or very similar *Wolbachia* that are present across the distribution of *N. vitripennis* (Raychoudhury et al., 2010). To compare supergroup‐specific effects, these two infections are separated into single *Wolbachia*‐infected wasp lines. A comparative analysis between the double‐infected, supergroup A‐infected, supergroup B‐infected, and uninfected lines reveal a consistent pattern of decreased longevity, quicker sperm depletion, and reduced family size for the infected individuals. While supergroup B infection shows a more pronounced negative effect on most of the traits investigated, supergroup A infection on the other hand shows milder negative effects only for some of those traits. By testing for differential titer of *Wolbachia* by qRT‐PCR, we also show a higher density of supergroup B‐ and double‐infected *Wolbachia* strains, compared with the supergroup A infection, across the majority of the developmental stages of *N. vitripennis*.

## 2 MATERIALS AND METHODS

### 2.1 *Nasonia vitripennis* lines used, their *Wolbachia* infections, and nomenclature

The *N. vitripennis* NV‐PU‐14 line was obtained from Mohali, Punjab, India, in 2015. NV‐PU‐14 was cured of *Wolbachia* by feeding the females with 1 mg/ml tetracycline in 10 mg/ml sucrose solution for at least two generations (Breeuwer & Werren, 1990). The curing was confirmed by PCR using supergroup‐specific *ftsZ* primers (Baldo et al., 2006), and CI crosses between the infected and uninfected lines. NV‐PU‐14 also served as the source strain for separating the two *Wolbachia* infections into single A and single B infected wasp lines.

To separate the *Wolbachia* supergroups, we utilized relaxed selection on the females by repeatedly mating them with uninfected males, which were obtained by antibiotic curing of the same NV‐PU‐14 line. Uninfected males do not have any sperm modification by *Wolbachia*, which results in the removal of any selection pressure on the females to maintain their *Wolbachia* infections. Repeated mating with uninfected males was continued for 10 generations till some of the progenies were found to be infected with either single A or single B supergroup infections. The single infection status of these *N. vitripennis* lines was confirmed by using supergroup‐specific *ftsZ* gene PCR primers (Baldo et al., 2006). The single infections were tested for CI phenotype. Single supergroup A *Wolbachia* infection lines showed incomplete CI while single supergroup B *Wolbachia* infection lines showed complete CI (Figure S1).

The preferred method of nomenclature of *Nasonia* lines and their *Wolbachia* infections includes information on supergroups and the host genotype. For example, [*w*NvitA *w*NvitB]V‐PU‐14 indicates that the host species is *N. vitripennis*, with NV‐PU‐14 as the host genotype, which has two *Wolbachia* infections, one each from supergroup A and supergroup B. However, since we used only *N. vitripennis* lines in this study, the nomenclature has been simplified by removing the species name. For example, the same double‐infected line will now be denoted as *w*A*w*B(PU), and when cured of these infections, as 0(PU). The single *Wolbachia*‐infected *N. vitripennis* lines used were designated as *w*A(PU) for the supergroup A‐infected line while *w*B(PU) for the supergroup B‐infected line. As the cured 0(PU) lines were in culture for 3 years, many of the infected lines were cured again to obtain “recently cured” lines to minimize the effects of any host divergence that might have accumulated within them. These “recently cured” lines were named 0(*w*A PU), 0(*w*B PU), and 0(*w*A*w*B PU).

Another *N. vitripennis* line, NV‐KA, obtained from Bengaluru, Karnataka, India, in 2016, was similarly named *w*A*w*B(KA). The MLST sequences of the two *Wolbachia* strains (one each from supergroups A and B), even in *w*A*w*B(KA), were found to be identical to *w*A*w*B(PU), and were also identical to all other *N. vitripennis* studied across the world (Prazapati, personal communication). *w*A*w*B(KA) was also cured of *Wolbachia* to obtain 0(*w*A*w*B KA).

All these wasp lines were raised on *Sarcophaga dux* fly pupae with a generation time of 14–15 days at 25°C, 60% humidity, and a continuous daylight cycle.

### 2.2 Sequential mating and sperm depletion of the males

To test the effect of *Wolbachia* on male reproductive traits like mating ability, individual males were assayed for the number of copulations they can perform and sperm depletion. As *N. vitripennis* is haplodiploid, every successful mating will result in both female and male progenies while an unsuccessful one will result in all‐male progenies. The males used were obtained from virgin females hosted with one fly pupa for 24 h and were not given any external sources of nutrition (usually a mixture of sucrose in water) before the experiment. Each male was then mated sequentially with virgin females from the same line. At the first sign of a male not completing the entire mating behavior (Jachmann & Assem, 1996), it was given a rest for half an hour and was subjected to mating again until it stopped mating altogether. The mated females were hosted after a day with one fly pupa for 24 h. The females were then removed, and the offsprings were allowed to emerge and then counted. The average number of copulations and the number of copulations before sperm depletion, were compared using the Kruskal–Wallis test with a significance level of .05. Mann–Whitney *U* test, with a significance level of .05, was used for comparisons between two groups.

### 2.3 Host longevity, family size, and fecundity

To test whether the presence of *Wolbachia* has any influence on longevity, emerging wasps of both sexes were kept individually in ria vials at 25°C, without any additional nutrition. Survival following emergence was measured by counting the number of dead individuals every 6 h. The Kaplan–Meier analysis, followed by log rank statistics, was used to identify differences between the strains with a significance level of .05.

To test for the effect of *Wolbachia* infections on the adult family size of virgin and mated females, each female was sorted at the pupal stage and separated into individual ria vials. To enumerate the brood size of mated females, some of these virgins were offered single males from the same line and observed till mating was successful. All the females were then hosted individually with one fly pupa for 24 h. These were kept at 25°C for the offspring to emerge, which were later counted for family size, by randomizing the ria vials in a double‐blind assay. The differences between groups were compared using the Kruskal–Wallis test with a significance level of .05. The Mann–Whitney *U* test, with a significance level of .05, was used to compare two groups.

To investigate whether *Wolbachia* affects the female fecundity, emerged females were hosted with one host for 24 h. The host pupa was placed in a foam plug, so that only the head portion of the pupa was exposed and available for the females to lay eggs. The females were removed after 24 h, and the eggs laid were counted under a stereomicroscope (Leica M205 C). The differences in fecundity were compared between groups using the Kruskal–Wallis test with a significance level of .05. The fecundity difference between two groups was compared using the Mann–Whitney *U* test with a significance level of .05.

### 2.4 Estimation of the relative density of *Wolbachia* infections across different developmental stages of *N. vitripennis*

To collect the different developmental stages, females were hosted for 4 h (instead of 24 h in the previous experiments), with one host, to narrow down the developmental stages of the broods. The larval and pupal stages (from day 3 to day 13 for males and from day 8 to day 14 for females) were collected every 24 h. Larval stages for females were not done to avoid any DNA contamination from the males as the two sexes are virtually indistinguishable at the larval stage. Three replicates of 10 larvae or pupae from the three strains, *w*A(PU), *w*B(PU), and *w*A*w*B(PU), were collected for each developmental stage. DNA extraction was done using the phenol‐chloroform extraction method, where samples were crushed in 200 μl of 0.5 M Tris‐EDTA buffer with 1% sodium dodecyl sulfate (SDS) and 2 μl of 22 mg/ml Proteinase K and incubated overnight at 37°C. DNA was purified with buffer saturated phenol and chloroform‐isoamyl alcohol solution (24:1) and precipitated overnight with isopropanol at −20°C. The precipitated DNA pellet was dissolved in 60 μl nuclease‐free water. The DNA concentration of the samples was measured using the Nanodrop 2000^®^ spectrophotometer (Thermo Scientific). The extracted DNA was checked with *28S* primers to confirm the PCR suitability of the DNA. The concentrations of all the samples were normalized to 200 ng/μl across the different male and female developmental stages, to be used for quantitative PCR. The CFX96 C1000® Touch Real‐time qRT‐PCR machine (BioRad) was used to assay the relative density of *Wolbachia* across the lines. Amplification was done for the *Wolbachia hcpA* gene (Forward Primer: 5′‐CTTCGCTCTGCTATATTTGCTGC‐3′, Reverse Primer: 5′‐CGAATAATCGCAACCGAACTG‐3′). The primers were tested to amplify both the *Wolbachia* supergroup A and B strains. *Nasonia S6K* was used as the control gene (Bordenstein & Bordenstein, 2011). Each reaction of 10 μl contained 5 μl of iTaq Universal SYBR^®^ Green supermix (BIORAD), .05 μl each of 10 μM of forward and reverse primers, and 200 ng of template DNA. Uninfected *N. vitripennis* DNA was used as negative control while DNase‐free water was used as a no‐template control. Reaction conditions included an initial denaturation step of 95°C for 3 min followed by 39 cycles of 95°C for 10 s, annealing, and amplification at 52°C for 30 s. All the reactions were performed in triplicates and included a melt curve, to check for nonspecific amplification. The relative *Wolbachia* density was estimated by calculating the mean delta threshold cycle (ΔC_q_), using the formula:

$$\mathrm{\Delta Cq}=\frac{1}{3}\sum_{j=1}^{3}\left[\frac{1}{3}\sum_{i=1}^{3}\text{hcpA}-\frac{1}{3}\sum_{i=1}^{3}S6K\right]$$

where *i*, number of technical replicates and *j*, number of biological replicates.

1/ΔCq was calculated and plotted to show the *Wolbachia* density across different developmental stages. The Mann–Whitney *U* test was used to compare two different lines with a significance level of .05.

## 3 RESULTS

### 3.1 The presence of *Wolbachia* reduces the life span of both males and females

**FIGURE 1:**
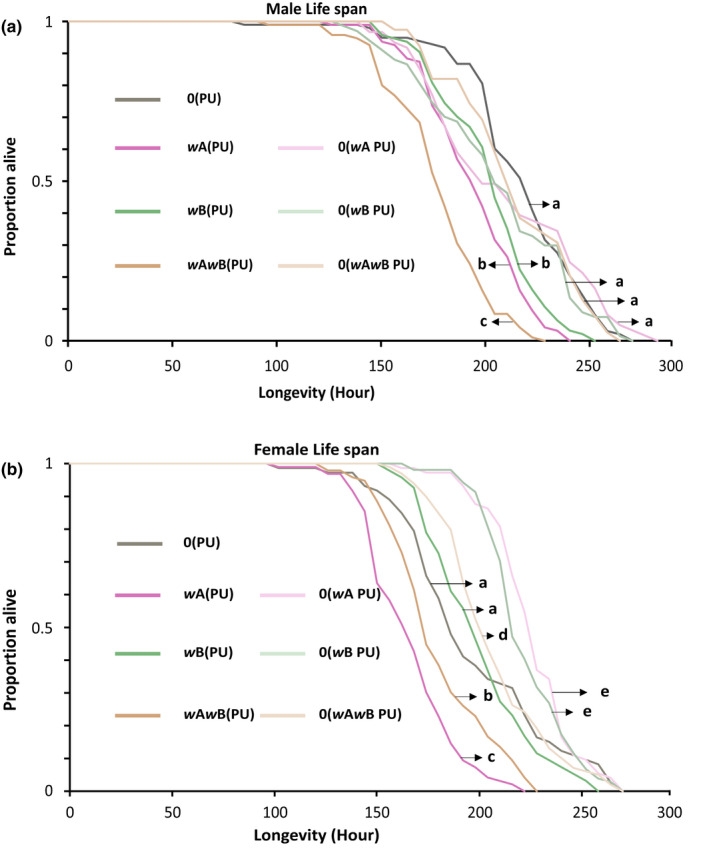
*Wolbachia*‐infected males and females show reduced life span. (a) Life span of males. (b) Life span of females. Statistical significance was tested using log rank statistics with *p* < .05.

*Wolbachia* can compete with the host for available nutrition, which can increase nutritional stress, resulting in a shortened life span for many hosts (Caragata et al., 2014; McMeniman et al., 2009). Therefore, we first investigated the effect of *Wolbachia* infections on the survival of both male and female wasps. As Figure 1(a), indicates, there is a significant difference in the life span of the infected males across the three infection types. The double‐infected line, *w*A*w*B(PU), starts to die off first and has a significantly shorter life span compared with the two single‐infected lines [log‐rank test, χ^2^ = 16.8, *p* < .001 for *w*A(PU) and χ^2^ = 33.9, *p* < .001 for *w*B(PU)]. Males from the uninfected line, 0(PU), lived the longest and showed significantly longer life span compared with all the other infected lines [log‐rank test: χ^2^ = 76.3, *p* < .001 for *w*A*w*B(PU); χ^2^ = 33.0, *p* < .001 for *w*A(PU); and χ^2^ = 16.3, *p* < .001 for *w*B(PU)]. However, there was no significant difference in the life span of the two single‐infected lines of *w*A(PU) and *w*B(PU) (log‐rank test, χ^2^ = 3.84, *p* = .05). Thus, the presence of *Wolbachia* leads to a significant reduction in the life span of the infected males. However, complex phenotypes like longevity can also be affected by the host genotype. Although all these four lines were derived from the same field‐collected isofemale line, continuous culturing in the laboratory can fix specific alleles within them resulting in inter‐line divergence. Moreover, it is also known that in *Nasonia,* the effect of *Wolbachia*‐induced phenotype is influenced by the hosts' genetic background (Raychoudhury & Werren, 2012). Therefore, we cured all these infections again and tested whether the host genotype, rather than *Wolbachia*, is causing this reduction in life span. This was done by comparing the life span of these newly cured lines back with the previously used uninfected line, 0(PU). The recently cured lines 0(*w*A PU), 0(*w*B PU), and 0(*w*A*w*B PU) showed significantly longer life span than their parental lines *w*A(PU) (log‐rank test: χ^2^ = 16.47, *p* < .0001), *w*B(PU) (log‐rank test: χ^2^ = 9.36, *p* < .01), and *w*A*w*B(PU) (log‐rank test: χ^2^ = 35.04, *p* < .0001), respectively, and were comparable with the uninfected line 0(PU) [log‐rank test: χ^2^ = 0.76, *p* = .38 for *w*A(PU), χ^2^ = 0.04, *p* = .8 and χ^2^ = 0.475, *p* = .50 for *w*A*w*B(PU)].

Similarly, infected females (Figure 1b) also showed a distinct reduction in life span when compared with the uninfected line. However, unlike the males, the single A‐infected *w*A(PU) females, showed the shortest life span [log‐rank test: χ^2^ = 11.2, *p* < .001 for *w*A*w*B(PU), χ^2^ = 56.9, *p* < .001 for *w*B(PU), and χ^2^ = 31.1, *p* < .001, for 0(PU)] followed by *w*A*w*B(PU) [log‐rank test: χ^2^ = 20.4, *p* < .001 for *w*B(PU) and χ^2^ = 12.9, *p* < .001 for 0(PU)]. Curiously, 0(PU) and *w*B(PU) females showed similar life spans (log‐rank test: χ^2^ = 0.24, *p* = .62).

The recently cured lines of 0(*w*A PU), 0(*w*B PU), and 0(*w*A*w*B PU) showed significant increase in the life span when compared to their parent lines *w*A(PU) (log‐rank test: χ^2^ = 107.31, *p* < .0001), *w*B(PU) (log‐rank test: χ^2^ = 39.02, *p* < .0001), and *w*A*w*B(PU) (log‐rank test: χ^2^ = 48.77, *p* < .0001), respectively. Surprisingly, the recently cured lines showed longer life span than 0(PU) [log‐rank test: χ^2^ = 19.31, *p* < .0001 for 0(*w*A PU), χ^2^ = 16.57, *p* < .0001 for 0(*w*B PU), and χ^2^ = 4.26, *p* < .05 for 0(*w*A*w*B PU)].

These results indicate a sex‐specific variation in longevity as the *w*A*w*B(PU) line shows the shortest life span among the males, but *w*A(PU) shows the shortest among the females. Moreover, the effect of single infections on longevity also varied among the sexes as *w*A(PU) and *w*B(PU) males had similar life spans, but it was *w*B(PU) and 0(PU) who had similar life spans among the females. But what is unambiguous from these results is that the uninfected line always lived the longest, irrespective of the sex of the host. The increase in the life span of the recently cured lines indicates that the presence of *Wolbachia* is associated with the reduction in life span and is thus costly for *N. vitripennis* to maintain.

### 3.2 The presence of *Wolbachia* reduces the number of copulations a male can perform

**FIGURE 2:**
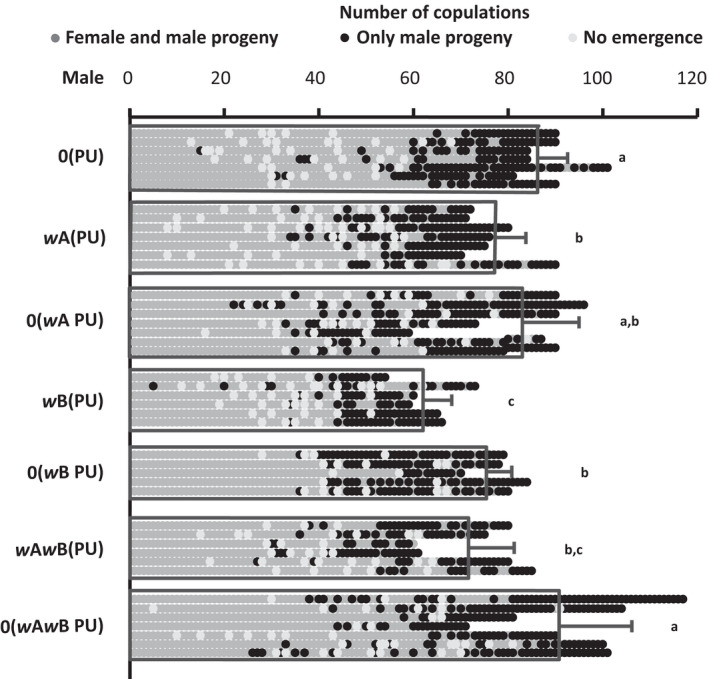
*Wolbachia*‐infected males show a reduction in the number of copulations. Males from different *Wolbachia* infection status strains were mated sequentially until each of them stopped mating. Some of the matings had “no emergence” of progenies because of poor host quality (shown by white dots). The results show that the presence of *Wolbachia* is associated with the reduction in the number of copulations a male can perform. The figure also shows whether the progenies of these sequential copulations produce any daughters or not, as a measure of sperm depletion. The details of sperm depletion are shown in Figure 3. Sample sizes for the strains 0(PU), *w*A(PU), 0(*w*A PU), *w*B(PU), 0(*w*B PU), *w*A*w*B(PU), and 0(*w*A*w*B PU) were *n* = 7, *n* = 7, *n* = 7, *n* = 6, *n* = 5, *n* = 6, and *n* = 7, respectively.

*Wolbachia* is known to be associated with a reduction in the number of mating a male can perform in *Ephestia kuehniella* (Sumida et al., 2017). To test whether similar effects are seen in *N. vitripennis*, we enumerated the number of copulations an individual male can perform across the infection types. As Figure 2 indicates, a significant difference was observed in the number of copulations performed by the males of different *N. vitripennis* lines (Kruskal–Wallis: H = 23.06, *p* < .001). There is indeed a reduction in the capacity of the infected males to mate. When compared with the uninfected line 0(PU), this reduction was most pronounced in *w*B(PU) (MWU, U = 30, *p* < .01), followed by *w*A*w*B(PU) and *w*A(PU), which showed similar successful copulations (MWU: U = 11, *p* = .49). The uninfected 0(PU) line produced males with the highest number of copulations [MWU: U = 32, *p* < .05 for *w*A(PU) and U = 27, *p* < .05 for *w*A*w*B(PU)]. Thus, the presence of *Wolbachia* substantially reduced the number of copulations that a male could perform. As Figure 2, indicates, males from most of the re‐cured lines showed a marked and significant increase in the number of copulations performed. This number in the re‐cured double‐infected line, 0(*w*A*w*B PU), increased to similar levels as shown by 0(PU) (an increase of 29%, MWU: U = 9.5, *p* = .2), while also showing a significant increase from its infected counterpart *w*A*w*B(PU) (from 73.5 ± 10.5 to 94.8 ± 15.39, MWU: U = 3, *p* < .05). Similarly, the number of copulations for the re‐cured single A supergroup‐infected line, 0(*w*A PU), also increased to the levels of the uninfected line 0(PU) (an increase of 7%, MWU: U = 20, *p* = .76). However, this increase (from 77.5 ± 6.3 to 83.5 ± 12.9) with its infected counterpart was not significant (MWU: U = 23, *p* = .48). The re‐cured line from the single B supergroup infection, 0(*w*B PU), was the only line that did not revert to uninfected levels (MWU: U = 22, *p* < .05) despite showing a marginal increase (from 62.8 ± 6.6 to 78.2 ± 5.1; MWU: U = 1, *p* < .05). However, what is evident is that the presence of *Wolbachia* is also associated with a reduction in the capability of a male to mate. Furthermore, by curing the infected lines again, we show that this decrease is not due to the host genotype but is an effect of the presence of *Wolbachia* in these lines.

### 3.3 *Wolbachia‐*infected males deplete their sperm reserves faster than the uninfected ones

**FIGURE 3:**
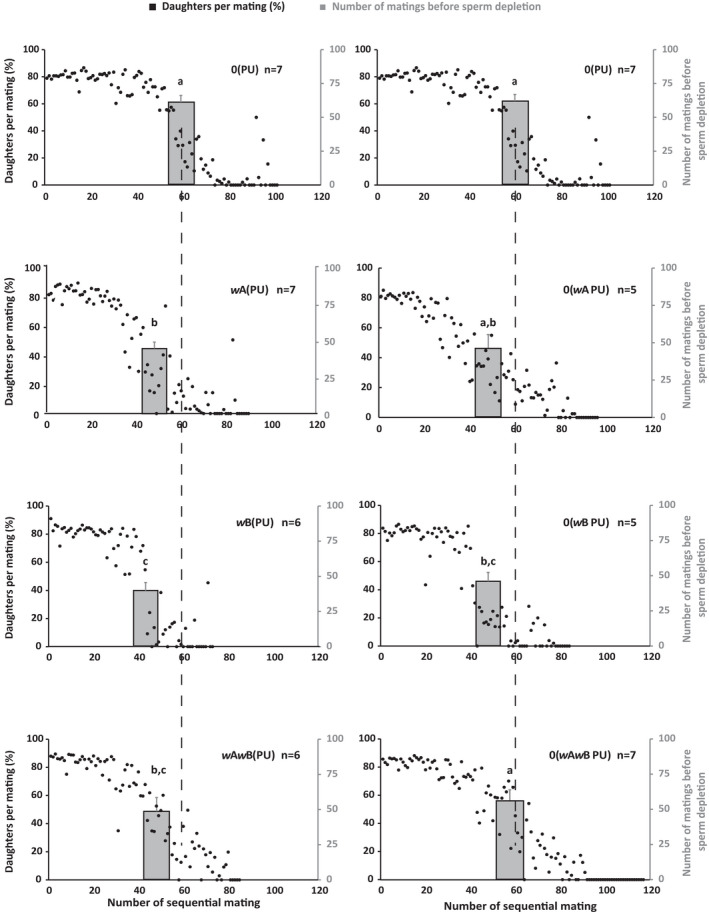
*Wolbachia‐*infected males deplete their sperm faster than the uninfected males. The Y‐axis in black on the left of each figure represents the percentage of daughters produced for each mating. The black dots represent the average number of daughters produced for each sequential mating by the males of different *Wolbachia* infection statuses (detailed in Figure 2). The number of daughters produced is taken as a measure of the number of sperm transferred during each mating. The Y‐axis, in gray, on the right, for each figure tallies the average number of copulations that yielded at least one daughter. Thus, it measures the number of mating before a male is depleted of its sperm. The left panel shows the males from *Wolbachia‐*infected lines, whereas the right panel shows their respective cured versions. Data for 0(PU) are repeated at the top for comparison. The statistical significance was tested using the Mann–Whitney *U* test with *p* < .05.

*Nasonia vitripennis* males are prospermatogenic (Boivin et al., 2005), where each male emerges with their full complement of mature sperm and has not been reported to produce any more during the rest of their life span (Chirault et al., 2016). Thus, if a single male is mated sequentially with as many females as it can mate with, it should eventually run out of this full complement of sperm and produce all‐male broods even after successful copulation. As Figure 2, indicates, each male did run out of sperm at the tail end of this continuous mating and produced only male progenies (shown by black dots). We looked at the number of mating done by these males before sperm depletion to see whether *Wolbachia* affects the sperm production in the males. As shown in Figure 3, the average number of daughter progenies reduced with the number of mating (shown by the primary Y‐axis on the left), indicating sperm depletion. Similar to copulation numbers, a significant difference was observed in the number of matings before sperm depletion between the males of different *Wolbachia*‐infected lines (Kruskal–Wallis: H = 21.48, *p* < .01). *w*B(PU) males were the quickest to deplete their sperm reserve [MWU: U = 27.5, *p* < .05 for *w*A(PU) and U = 30, *p* < .01 compared for 0(PU)]. This was followed by *w*A*w*B(PU) and *w*A(PU) (MWU: U = 13, *p* = .7). However, the uninfected males from 0(PU) were the slowest to deplete their sperm reserve [MWU: U = 35, *p* < .01 for *w*A(PU) and U = 24, *p* < .05 for *w*A*w*B(PU)]. We again tested whether the host genotype, rather than *Wolbachia*, is causing this rate of sperm depletion, by comparing it with the recently cured lines. As shown in Figure 3, the number of mating before sperm depletion increased for the recently cured 0(*w*A PU) line up to the levels of 0(PU) (an increase of 5%, MWU: U = 30, *p* = .06). However, this increase (from 48.14 ± 4.94 to 50.57 ± 9.41) was not significantly different from the infected counterpart *w*A(PU) (MWU: U = 16, *p* = .8). Rates of depletion for 0(*w*A*w*B PU) also increased up to the levels of 0(PU) (an increase of 15.2%, MWU: U = 21, *p* = .66). Again, the recently cured line 0(*w*B PU), increased from *w*B(PU) (from 41 ± 1.67 to 47.6 ± 6.0, MWU: U = 0, *p* < .05) but was still lower than 0(PU) (MWU: U = 23, *p* < .05). These results indicate that the presence of *Wolbachia* has a significant negative impact on the number of sperm produced or utilized by the infected males.

### 3.4 *Wolbachia‐*infected females produce fewer offspring

*Wolbachia* is known to have a negative impact on the progeny family size of its host (Hoffmann et al., 1990; Hohmann et al., 2001). To test whether a similar effect is seen in *N. vitripennis*, we enumerated the family sizes for both virgin and mated females for the four different *Wolbachia*‐infected lines and their recently cured counterparts.

**FIGURE 4:**
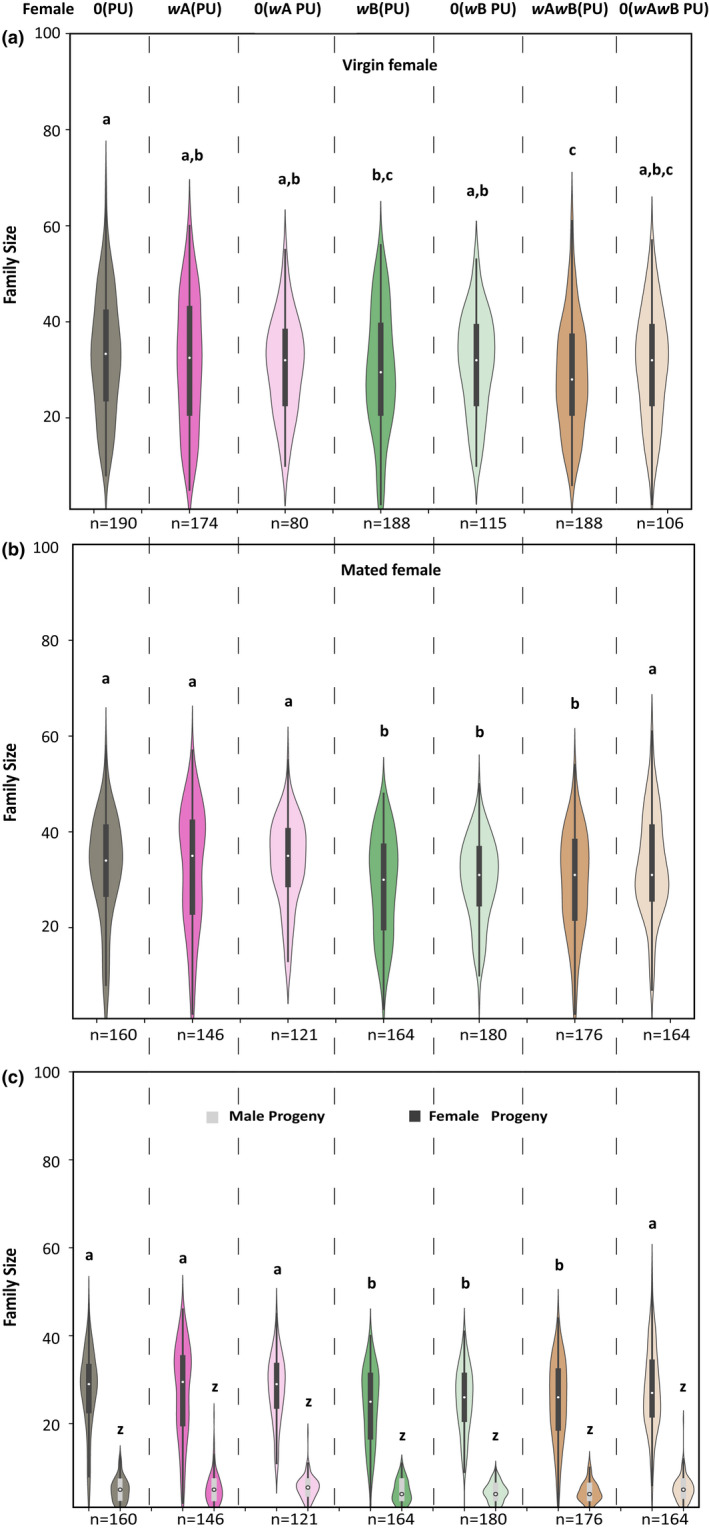
*Wolbachia*‐infected females produce fewer offspring. The family size is produced by females when hosted as virgins (a) and mated (b). The difference in the family size of mated females is due to the difference in the number of daughters (c) as there is no significant difference in the number of males produced. The statistical significance was tested using the Mann–Whitney *U* test with *p* < .05.

As Figure 4(a), indicates, there is a significant reduction in the average family sizes of all‐male broods produced by the virgin females of the *Wolbachia*‐infected *N. vitripennis* lines (Kruskal–Wallis: H = 12.6, *p* < .05). When compared with the uninfected line 0(PU), this reduction was most pronounced in *w*A*w*B(PU) (MWU: U = 21,151.5, *p* < .01) followed by *w*B(PU) (MWU: U = 19,880.5, *p* < .05). *w*B(PU) and *w*A*w*B(PU) showed similar family sizes (MWU: U = 18,582.5, *p* = .29). However, *w*A(PU) produced similar family sizes when compared with 0(PU) (MWU: U = 17,191, *p* = .39) and *w*B(PU) (MWU: U = 17,284, *p* = .26) but had larger all‐male brood sizes than *w*A*w*B(PU) (MWU: U = 18,252, *p* < .05). We also compared the recently cured single and double‐infected lines with the infected parental lines. 0(*w*B PU) and 0(*w*A*w*B PU) showed marginal increase in their family sizes, which was comparable with the uninfected line 0(PU) [an increase of 1.5%, MWU: U = 11,554, *p* = .29 for 0(*w*B PU); an increase of 2%, MWU: U = 10,798, *p* = .21 for 0(*w*A*w*B PU)]. However this marginal increase [from 29.84 ± 12.94 to 31.44 ± 10.30 for 0(*w*B PU) and from 28.99 ± 11.60 to 30.97 ± 11.48 for 0(*w*A*w*B PU)] was not significantly different from their infected counterparts *w*B(PU) (MWU: U = 9963.5, *p* = .34) and *w*A*w*B(PU) (MWU: U = 8650.5, *p* = .09), respectively. The recently cured line 0(*w*A PU) did not show any increase in the family size and was comparable with *w*A(PU) (MWU: U = 7085.3, *p* = .63) and 0(PU) (MWU: U = 8161, *p* = .22).

Similar to the virgin females, a reduction was also observed in average family sizes of mated females of the infected lines as shown in Figure 4(b) (Kruskal–Wallis: H = 30.45, *p* < .0001). When compared with the uninfected line 0(PU), this reduction is most pronounced in *w*B(PU) (MWU: U = 15,582, *p* < .01) and *w*A*w*B(PU) (MWU: U = 16,303, *p* < .01). However, *w*B(PU) and *w*A*w*B(PU) showed similar family sizes (MWU: U = 13,732.5, *p* = .55). Interestingly, the *w*A(PU) line showed similar family sizes as 0(PU) (MWU: U = 11,396.5, *p* = .86) but had larger family sizes when compared to *w*B(PU) (MWU: U = 14,080, *p* < .01) and *w*A*w*B(PU) (MWU: U = 14,682, *p* < .05). Upon curing, the average family sizes of the recently cured 0(*w*A*w*B PU) reverted back to the levels of the uninfected line 0(PU) (an increase of 11.8%, MWU: U = 13,295, *p* = .61) showing a significant increase from the infected counterpart *w*A*w*B(PU) (from 29.49 ± 10.67, MWU: U = 12,023, *p* < .05). The recently cured line 0(*w*A PU) did not show any significant increase from the infected counterpart *w*A(PU) (MWU: U = 8385.5, *p* = .69) and was comparable with 0(PU) (MWU: U = 9022.5, *p* = .5). However, the 0(*w*B PU) line did not show an increase up to the levels of the uninfected line 0(PU) (an increase of 4.3%, MWU: U = 16,782, *p* < .05). The marginal increase in the family sizes (from 28.72 ± 10.46 to 29.97 ± 8.59) was not significantly different from the parental line *w*B(PU) (MWU: U = 13,854, *p* = .47).

To understand whether this difference in the family size of the mated females is due to the production of fewer daughters or sons or both, we compared their numbers separately for the different infection lines (Figure 4c). No difference was observed in the number of sons produced by the mated females. However, significant differences were observed in the number of daughters produced. When compared to the uninfected line 0(PU), *w*B(PU), and *w*A*w*B(PU) showed the least number of daughters produced [MWU: U = 15,964, *p* < .01 for *w*B(PU) and U = 16,283, *p* < .01 for *w*A*w*B(PU)] whereas *w*B(PU) and *w*A*w*B(PU) produced nearly equal number of daughters (MWU: U = 13,392, *p* = .33). Again, *w*A(PU) line produced equal number of daughters compared with 0(PU) (MWU: U = 11,543, *p* = .98) but higher in number than *w*B(PU) and *w*A*w*B(PU) [MWU: U = 14,201, *p* < .01 for *w*B(PU) and MWU: U = 14,372, *p* < .05 for *w*A*w*B(PU)]. Upon curing, the recently cured 0(*w*A*w*B PU) reverted to the levels of the uninfected line 0(PU) (MWU: U = 13,545, *p* = .42) showing a significant increase in the number of daughters from the infected counterpart *w*A*w*B(PU) (MWU: U = 12,331, *p* < .039). The recently cured line 0(*w*A PU) did not show any increase in the number of daughters produced from their infected counterpart *w*A(PU) (MWU: U = 8468 *p* = .79) and was also comparable with 0(PU) (MWU: U = 9330, *p* = .84). However, recently cured line 0(*w*B PU) did not increase to the levels of the uninfected line 0(PU) (MWU: U = 16,749.5, *p* < .01).

To determine whether the negative effect on progeny family size in females is not limited to NV‐PU‐14 *N. vitripennis*, we also checked the virgin and mated female progeny family size of another *N. vitripennis* line NV‐KA from Bengaluru (India). The double‐infected *w*A*w*B(KA) line was cured to generate recently cured 0(*w*A*w*B KA). In the average family sizes of all‐male broods produced by the virgin females (Figure S3a), the recently cured line 0(*w*A*w*B KA) has more progenies as compared to *w*A*w*B(KA) (MWU: U = 1534.5, *p* < .05). Similar to the virgin females, the mated females (Figure S3b) of 0(*w*A*w*B KA) also produced more progenies as compared to *w*A*w*B(KA) (MWU: U = 2568.5, *p* < .05). Thus, the negative effects of the presence of *Wolbachia* on the family sizes produced were confirmed in two different geographical lines of *N. vitripennis*.

### 3.5 *Wolbachia* negatively impacts the fecundity of infected females

**FIGURE 5:**
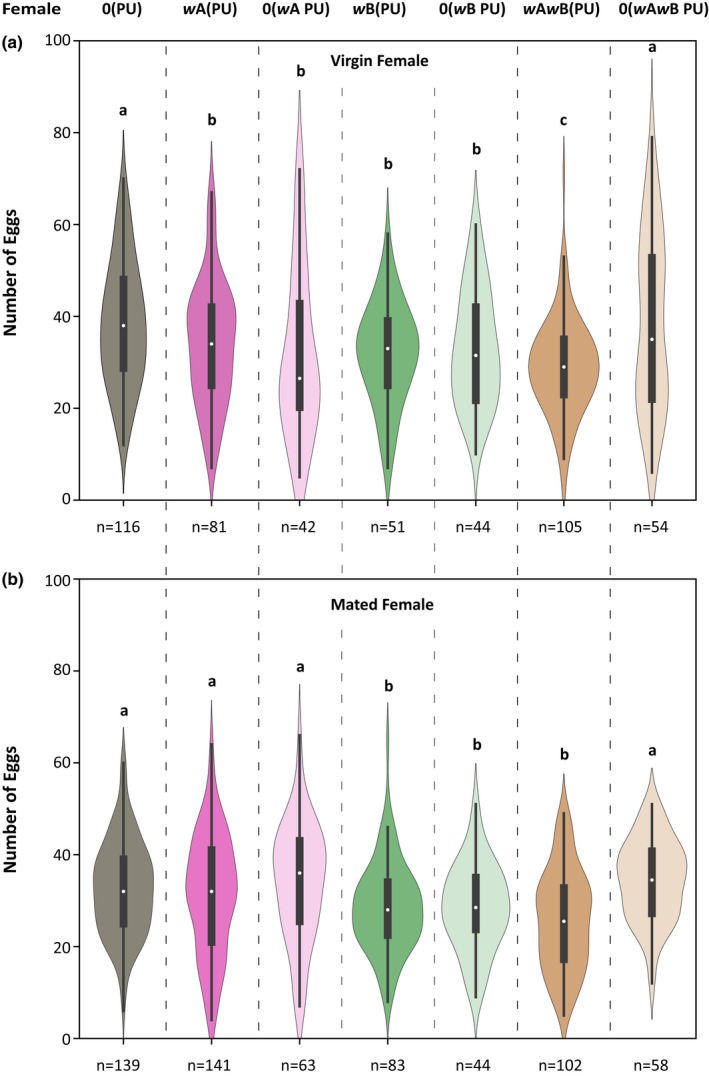
*Wolbachia* infection reduces female fecundity. The measure of fecundity (number of eggs laid) by females of different *Wolbachia* infection status [virgin females (a) and mated females (b)]. The statistical significance was tested with the Mann–Whitney *U* test, *p* < .05.

To check whether the differences in the family sizes between the different infected lines of *N. vitripennis* were due to the number of eggs being laid by the females, we looked at the fecundity of both virgin and mated females across these lines. Among the virgin females (Figure 5a) significant differences were observed in the fecundity of the different *N. vitripennis* lines (Kruskal–Wallis: H = 28.8, *p* < .001). *w*A*w*B(PU) had the least fecundity (MWU: U = 8424.5, *p* < .0001) when compared to the uninfected line 0(PU). Significant differences were observed between 0(PU) and *w*A(PU) (MWU: U = 5383, *p* < .05), between 0(PU) and *w*B(PU) (MWU: U = 3600.5, *p* < .05), and also between *w*A(PU) and *w*A*w*B(PU) (MWU: U = 5155, *p* < .01). However, no difference was observed between *w*A(PU) and *w*B(PU) (MWU: U = 2153.5, *p* = .46). Upon curing, the recently cured 0(*w*A*w*B PU) reverted to the levels of the uninfected line 0(PU) (MWU: U = 3404.5, *p* = .42) showing a significant increase in fecundity from the infected counterpart *w*A*w*B(PU) (MWU: U = 2101.5, *p* < .05). However, the recently cured line 0(*w*A PU) (MWU: U = 3137.5, *p* < .01) and 0(*w*B PU) (MWU: U = 3077, *p* < .05) did not increase to the levels of the uninfected line 0(PU).

Significant differences were also observed in the number of eggs laid by the mated females (Figure 5b) (Kruskal–Wallis: H = 42.6, *p* < .001). *w*A*w*B(PU) again had the least fecundity (MWU: U = 9410.5, *p* < .0001) when compared to the uninfected line 0(PU). *w*B(PU) had similar fecundity as that of *w*A*w*B(PU) (MWU: U = 4731, *p* = .098) but had significantly lower fecundity than 0(PU) (MWU: U = 7052.5, *p* < .01) and *w*A(PU) (MWU: U = 6684, *p* < .05). However, *w*A(PU) showed higher fecundity than *w*A*w*B(PU) (MWU: U = 8899, *p* < .01) and was similar to the uninfected line 0(PU) (MWU: U = 10,100, *p* = .5). Upon curing, the recently cured 0(*w*A*w*B PU) line reverted to the levels of the uninfected line 0(PU) (MWU: U = 3415, *p* = .149), showing a significant increase in fecundity from the infected counterpart *w*A*w*B(PU) (MWU: U = 1265, *p* < .0001). However, the recently cured line 0(*w*B PU) (MWU: U = 3655, *p* < .05) did not increase to the levels of the uninfected line 0(PU) and was still comparable with the infected counterpart *w*B(PU) (MWU: U = 1713.5, *p* = .79)

The results thus suggest a negative effect of *Wolbachia* on egg production in females. The assay also established that the difference in family sizes can be due to the differences in the fecundity of the females.

### 3.6 Relative *Wolbachia* density in single and multiple *Wolbachia* infections *N. vitripennis* lines

**FIGURE 6:**
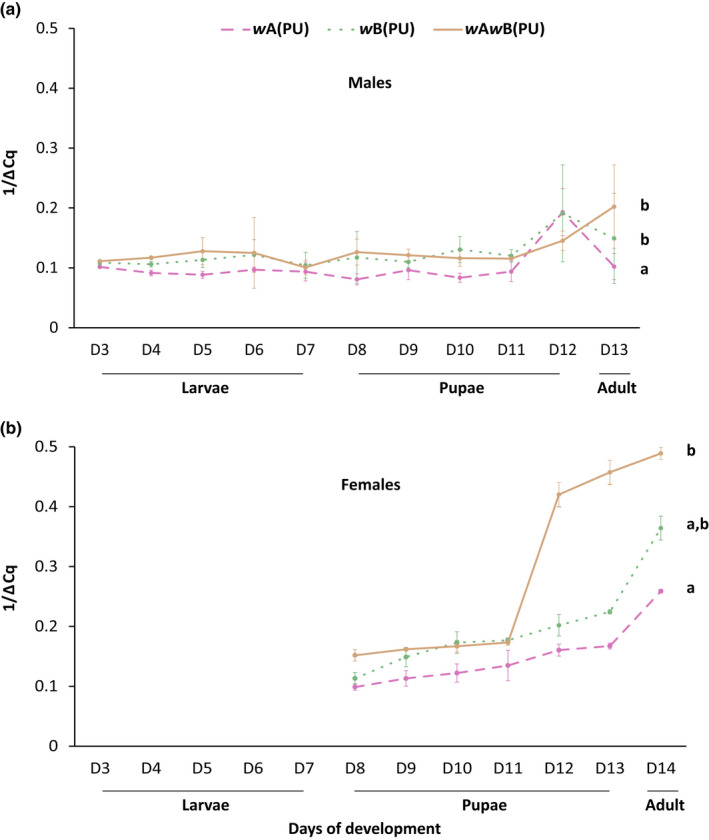
Quantitative estimation of *Wolbachia* across different developmental stages of *N. vitripennis* males (a) and females (b). The statistical significance between groups was tested using the Mann–Whitney *U* test, *p* < .05.

*Wolbachia* density has a major role to play in expressing the effects of the infection on host biology (Hoffmann et al., 1996; Min & Benzer, 1997). An increase in cellular *Wolbachia* density is often associated with a greater expression of their effects (Breeuwer & Werren, 1993). Thus, we estimated *Wolbachia* titers across the different developmental stages of *N. vitripennis*. In the case of males (Figure 6a) *w*A(PU) had the lowest *Wolbachia* density across the different larval and pupal developmental stages when compared with *w*B(PU) (MWU: U = 11, *p* < .01) and *w*A*w*B(PU) (MWU: U = 12, *p* < .01). However, no such differences were found between *w*B(PU) and *w*A*w*B(PU) (MWU: U = 51, *p* = .56).

In the case of females (Figure 6b), *w*A(PU) showed lower levels of *Wolbachia* when compared to *w*A*w*B(PU) (MWU: U = 8, *p* < .05) again at the pupal and adult stages. However, no difference was observed between *w*A(PU) and *w*B(PU) (MWU: U = 12, *p* = .12) and also *w*B(PU) and *w*A*w*B(PU) (MWU: U = 19, *p* = .5).

## 4 DISCUSSION

The results from this study (summarized in Table 2) demonstrate a sex‐independent cost of the presence of single and multiple *Wolbachia* infections. Many phenotypes show a significant reduction across the sexes such as longevity (Figure 1), where the infected males and females show reduced life span. When compared with the uninfected line 0(PU), the *Wolbachia*‐infected lines *w*B(PU) and *w*A*w*B(PU) have reduced life spans. However, sex‐specific variations have also been observed among the infected lines where *w*A(PU), in females, had the shortest life span, while in the case of males, had a greater life span than that of *w*A*w*B(PU).

**TABLE 2:** Effect of *Wolbachia* infections on *Nasonia vitripennis* (Summary)

Phenotype	Host sex	Effect of *Wolbachia*	“Cost” compared with 0(PU)
Life span	Male	0(PU) > *w*B(PU) = *w*A(PU) > *w*A*w*B(PU)	*w*A(PU) = 11.1%, *w*B(PU) = 6.5%, *w*A*w*B(PU) = 15.5%
	Female	0(PU) = *w*B(PU) > *w*A*w*B(PU) > *w*A(PU)	*w*A(PU) = 17.7%, *w*A*w*B(PU) = 15.5%
Number of copulations	Male	0(PU) > *w*A(PU) *w*B(PU) and *w*A*w*B(PU) *w*A(PU) = *w*A*w*B(PU), *w*A(PU) > *w*B(PU), *w*A*w*B(PU) = *w*B(PU)	*w*A(PU) = 12.4%, *w*B(PU) = 28.8%, *w*A*w*B(PU) = 16.7%
Sperm depletion	Male	0(PU) > *w*A(PU) *w*B(PU) and *w*A*w*B(PU) *w*A(PU) = *w*A*w*B(PU), *w*A(PU) > *w*B(PU), *w*A*w*B(PU) = *w*B(PU)	*w*A(PU) = 19.4%, *w*B(PU) = 31.3%, *w*A*w*B(PU) = 15.7%
Progeny family size	Female		
	a. Virgin	0(PU) > *w*B(PU) and *w*A*w*B(PU), 0(PU) = *w*A(PU), *w*A(PU) > *w*A*w*B(PU), *w*A(PU) = *w*B(PU), *w*B(PU) = *w*A*w*B(PU)	*w*B(PU) = 10%, *w*A*w*B(PU) = 12.4%
	b. Mated	0(PU) = *w*A(PU) > *w*A*w*B(PU) = *w*B(PU)	*w*B(PU) = 11.5%, *w*A*w*B(PU) = 9.1%
Fecundity	Female		
	a. Virgin	0(PU) > *w*A(PU) = *w*B(PU) > *w*A*w*B(PU)	*w*A*w*B(PU) = 14.1%
	b. Mated	0(PU) = *w*A(PU) > *w*B(PU) = *w*A*w*B(PU)	*w*B(PU) = 9.1%, *w*A*w*B(PU) = 18.2%
*Wolbachia* density	Male	*w*A*w*B(PU) = *w*B(PU) > *w*A(PU)	
	Female	*w*A*w*B(PU) > *w*A(PU), *w*A*w*B(PU) = *w*B(PU)	

*Wolbachia* affects the reproductive capabilities of the infected males, reduces their copulation capability (Figure 2), and also leads to quicker sperm depletion (Figure 3). Such negative effects on reproductive traits were also observed in females, where the infected females produce fewer progenies (Figure 4). These differences are elicited at the level of female fecundity, where the infected females lay fewer eggs (Figure 5), indicating that the negative effects of *Wolbachia* manifest themselves even before the egg‐laying stage. However, the egg to larval to pupal stage mortality could also have an effect on the brood sizes, but these were not assayed.

In most cases, these negative effects disappear with the removal of *Wolbachia*, indicating the role of *Wolbachia* in producing these negative effects and not the host genotype. In phenotypes like longevity, family sizes, and fecundity, the recently cured lines show a significant increase, suggesting that the negative effects are due to the presence of *Wolbachia*. However, 0(*w*B PU) did not revert to the levels of 0(PU) in the number of copulations performed, sperm depletion assay, and female fecundity. A possible reason could be some residual effects of the parent genotype in 0(*w*B PU) but needs further empirical validation.

Our experiments indicate an additive or synergistic effect of the presence of the two different *Wolbachia* supergroups in the double‐infected line *w*A*w*B(PU). Evidence of such effects can be seen in traits like male longevity (additive effect) where the deficit in longevity for *w*A*w*B(PU) is equal to the total deficits caused by *w*A(PU) and *w*B(PU). Similarly, for traits like female longevity, virgin female family size, and female fecundity, the negative effects on the *w*A*w*B(PU) line appear to be a combined effect of both the *w*A(PU) and *w*B(PU) lines (i.e., a synergistic effect). Since the two supergroup infections are bidirectionally incompatible with each other, it is plausible that they are also competing for the host nutrition, which can further enhance the negative impacts of these infections.

Our results also demonstrate supergroup‐specific negative effects on the host. Supergroup B *Wolbachia* is costlier to maintain in both sexes than supergroup A. While the *w*B(PU) line shows strong effects of supergroup B *Wolbachia* on all the traits studied across the sexes, *w*A(PU) has significant negative effects of supergroup A *Wolbachia* only on the reproductive traits of the males and the longevity of females. *w*A(PU) females, as an exception, have their family sizes comparable with 0(PU). These observations are unique as no comprehensive data are available on the supergroup‐specific cost of *Wolbachia* infections in most insect systems.

Previous reports have suggested a direct correlation between *Wolbachia* density and the level of CI (Breeuwer & Werren, 1993; Dutton & Sinkins, 2004; Ikeda et al., 2003; Noda et al., 2001; Ruang‐Areerate & Kittayapong, 2006). Our results also suggest that the cost of *Wolbachia* maintenance is correlated with the density of *Wolbachia* strains present in the host. Thus, in the case of females, *w*A*w*B(PU), which shows a high bacterial load, has reduced fecundity and longevity. Similarly, in the case of males, the *w*A*w*B(PU) shows a reduced number of copulations and the number of sperm produced/transferred. *w*B(PU), shows substantial detrimental effects in both the sexes of the host, which is similar to *w*A*w*B(PU). This again can be explained by the high *Wolbachia* supergroup B load in both the sexes of *w*B(PU). However, although *w*A(PU) males show reduced number of copulations and number of sperm produced/transferred, *w*A(PU) females had progeny family sizes and the fecundity of mated females comparable with 0(PU) females. A possible explanation for this can be the relatively low density of supergroup A *Wolbachia* in *w*A(PU) across the different developmental stages (Figure 6) as compared to the other infections. Supergroup A‐infected *N. vitripennis* lines are known to have relatively higher levels of phage density (Bordenstein et al., 2006), and according to the phage density model, this higher phage density has an inverse impact on the level of CI caused by supergroup A *Wolbachia*. This results in a significant reduction in the *Wolbachia* titer and hence shows a milder intensity of the effect of CI. Our results also confirm these previous reports of the positive correlation between *Wolbachia* abundance and the level of CI induced not only in *N. vitripennis* (Bordenstein et al., 2006) but also in other insect taxa as well (Ijichi et al., 2002; Kondo et al., 2002). *w*B(PU) *Wolbachia* shows complete CI and has higher *Wolbachia* titers in both males and females, which is also comparable with the *Wolbachia* densities of *w*A*w*B(PU) males and females, whereas *w*A(PU) has the lowest *Wolbachia* titers among the three strains and shows incomplete CI (Figure S1). Thus, higher levels of *Wolbachia* in *w*B(PU) than in *w*A(PU) can also explain the more severe effects in *w*B(PU) than *w*A(PU).

The negative fitness effects of CI‐inducing *Wolbachia*, and nutritional competition raises important questions on the maintenance of these endosymbionts over long evolutionary time scales. Theoretical studies indicate that evolution towards mutualism can aid the long‐term persistence of these maternally inherited reproductive parasites (Prout, 1994; Turelli, 1994). Moreover, if there are indeed some adverse effects of maintaining *Wolbachia*, then hosts would be under strong selection pressure to develop immunity against them. Evidence suggests that there are examples of such emergence of host genetic factors against *Wolbachia* infections in *Drosophila* and mosquitoes (Zug & Hammerstein, 2015). Host suppressor alleles have been identified, which confer resistance against feminizing (Rigaud et al., 1999) and male‐killing *Wolbachia* (Hornett et al., 2006). However, no such host genetic factors have been found for CI‐inducing *Wolbachia*, especially in *N. vitripennis*. Therefore, a possible explanation for the maintenance of these multiple infections then comes from the high efficiency of transmission of these infections in *N. vitripennis*, which is nearly 100% (Breeuwer & Werren, 1990). Theoretical studies also suggest that even in the presence of selective pressures, multiple infections are maintained and transmitted owing to the fitness advantages conferred and CI (Vautrin et al., 2008).

Another possibility can be that these *Wolbachia* infections in *N. vitripennis* are relatively recent, the evidence of which comes from the rapid spread of *Wolbachia* in populations of *N. vitripennis* across North America and Europe (Raychoudhury et al., 2010). These recent infections, although bearing a cost on the host at present, might eventually lead to the evolution of host resistance against them.

Our results indicate supergroup B to be a “stronger” *Wolbachia* than supergroup A and any competition for nutritional resources and niche habituation between them should drive out supergroup A *Wolbachia*. Moreover, *w*A(PU) has milder effects on females with the reduction in longevity being the only pronounced negative effect. Therefore, the continuation of this supergroup infection is difficult to explain. One possibility could be the supergroup A infection conferring mutualistic effects on the host. This strain is closely related to other supergroup A *Wolbachia* strains like *w*Mel in *D. melanogaster* and *w*Ha, *w*Au, and *w*Ri in *D. simulans* (Díaz‐Nieto et al., 2021). *w*Mel in *D. melanogaster* and *w*Ha, *w*Au, and *w*Ri in *D. simulans* are known to provide defense against viral infections to their hosts (Bhattacharya et al., 2017; Pimentel et al., 2021; Teixeira et al., 2008). The continued presence of supergroup A *Wolbachia* in *N. vitripennis* could be due to such defenses against viral infections, but this hypothesis remains to be tested.

The higher cost of maintenance of supergroup B *Wolbachia* can be an attribute of the CI phenotype induced by supergroup B *Wolbachia*. Complete CI (i.e., nearly 100%) are rare events reported mainly for supergroup B *Wolbachia* in *Culex pipiens*, *Aedes aegypti* (Sinkins et al., 2005; Xi et al., 2005), and *N. vitripennis* (Figure S1 and Bordenstein et al., 2006). This essentially means that nearly the entire sperm complement of each male has the *Wolbachia*‐induced CI modification, and correspondingly, nearly all the eggs from the females have the rescue effect (Werren et al., 2008). Introducing 100% modification and rescue would necessitate relatively high *Wolbachia* titers to be maintained in both sexes, which in turn can cause an elevated nutritional burden, eventually resulting in negative effects on the physiological traits of the host. This seems a plausible explanation for both the high negative effects and the relatively higher titers of *Wolbachia* seen in *w*B(PU).

CI‐inducing *Wolbachia* is known to have negative effects on various physiological traits in the vast majority of its host population (summarized in Table 1). The present study also suggests such effects, or a “cost,” associated with the maintenance of *Wolbachia* infection in *N. vitripennis*. This is in contrast to the previous reports suggesting positive fitness effects (Stolk & Stouthamer, 1996) and no fitness effects (Bordenstein & Werren, 2000) of *Wolbachia* on *N. vitripennis*. However, the strain used are all from India and the negative effects seen can be unique to these lines. Although the lines used here have the same or very similar *Wolbachia* as far as sequence uniformity is concerned across the five MLST alleles, other lines from other continents need to be analyzed to confirm whether this effect is ubiquitous in *N. vitripennis*.

## AUTHOR CONTRIBUTIONS

**Alok Tiwary:** Conceptualization (lead); data curation (lead); formal analysis (lead); investigation (lead); methodology (lead); visualization (lead); writing – original draft (lead); writing – review and editing (lead). **Rahul Babu:** Data curation (supporting). **Ruchira Sen:** Formal analysis (supporting); visualization (supporting). **Rhitoban Raychoudhury:** Conceptualization (supporting); funding acquisition (lead); supervision (lead); visualization (supporting); writing – original draft (supporting); writing – review and editing (supporting).

## CONFLICT OF INTEREST

The authors declare no conflict of interest.

## Supporting information


Figure S1‐S3

## Data Availability

The raw data for all the experiments have been archived at Dryad with https://doi.org/10.5061/dryad.w0vt4b8s9, https://datadryad.org/stash/share/gKvBkEcYjmrNULX4F53Ktu5Pt‐P1pcXBnbA5Ib_HEPo.
