# Supplementary material for: Bacterial supergroup‐specific “cost” of Wolbachia infections in Nasonia vitripennis
Source: Ecol Evol. 2022 Sep 13;12(9):e9219. doi: 10.1002/ece3.9219 (PMC9468909; doi:10.1002/ece3.9219)
Supplement: Supplementary file 1 — Figure S1‐S3 [file ECE3-12-e9219-s001.docx]

Supplementary Information for Publication

**Bacterial supergroup specific “Cost” of *Wolbachia* infections in *Nasonia vitripennis***

Alok Tiwary^1^, Rahul Babu^1, 2^, Ruchira Sen^3^, Rhitoban Raychoudhury^1*^

1. Department of Biological Sciences, Indian Institute of Science Education and Research (IISER) Mohali, Knowledge City, Sector 81, SAS Nagar, Manauli, PO 140306, Punjab, India.
2. Zoological Survey of India, Kolkata, West Bengal-700053, India
3. Guru Gobind Singh College, Sector 26, Chandigarh-160019, India.

*Corresponding author: Rhitoban Raychoudhury, Department of Biological Sciences, Indian Institute of Science Education and Research (IISER) Mohali, Sector 81, Knowledge City, SAS Nagar, Manauli, PO 140306 (Punjab), India

Email: rhitoban@iisermohali.ac.in


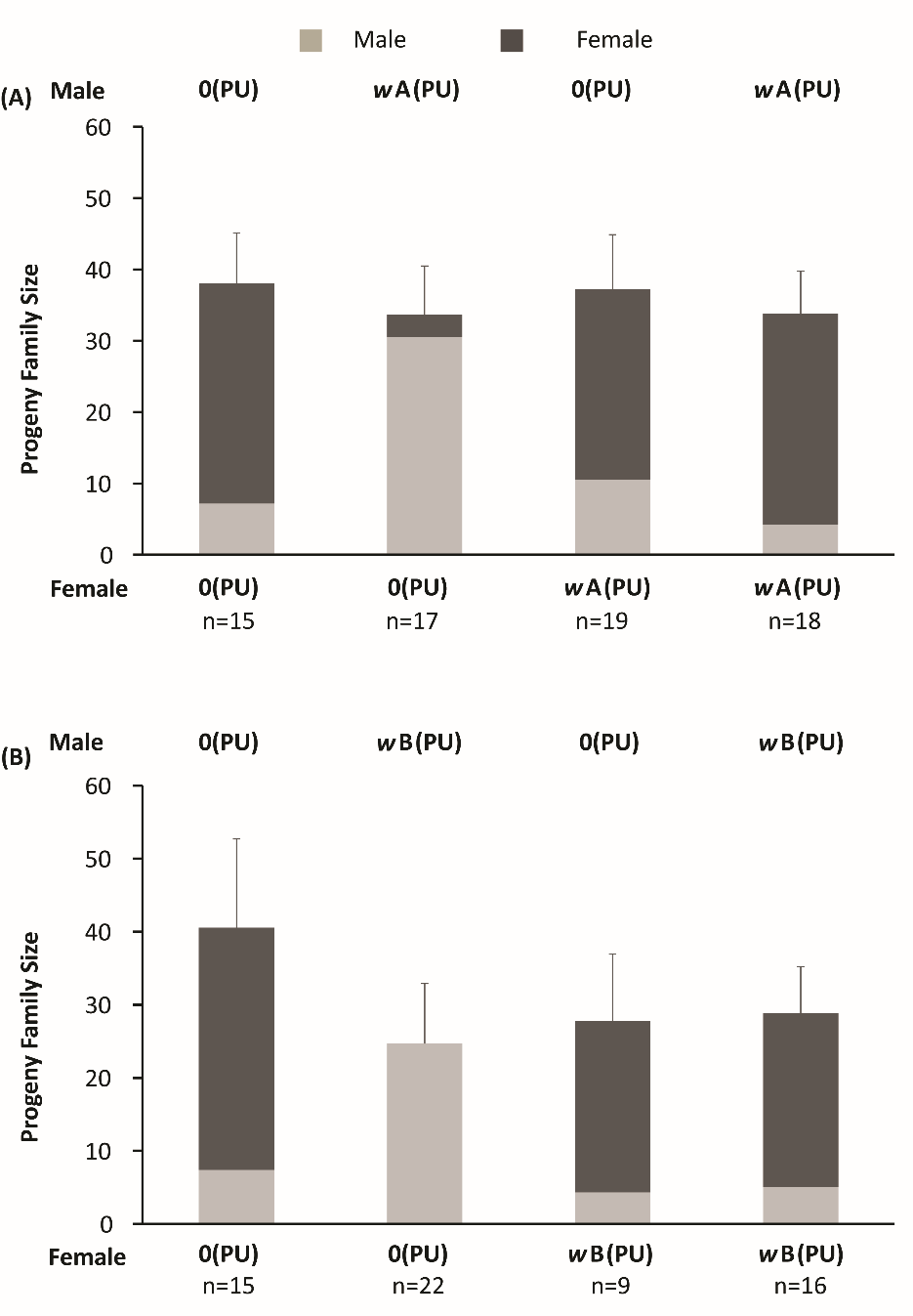


**Figure S1. CI crosses for single *Wolbachia* infection strains (A) *w*A(PU), and (B) *w*B(PU)**

*w*A(PU) males show incomplete CI with 0(PU) females as the cross produces female progenies as well (A). *w*B(PU) males show complete CI with 0(PU) females which leads to an all-male brood (B).


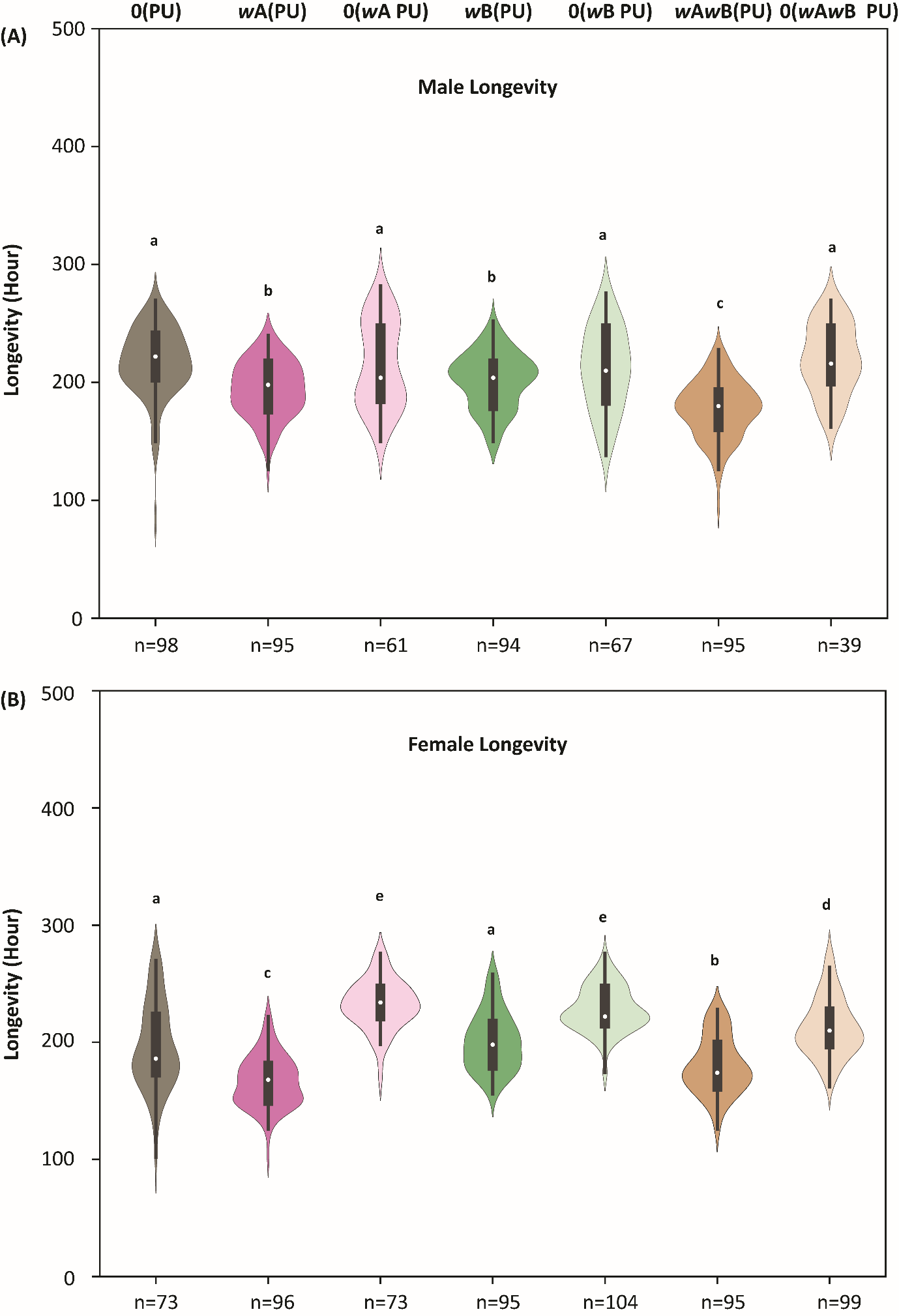


**Figure S2. The average life span and distribution for (A) males and (B) females**.

1. A significant difference was observed between the average life span of the males of different *N. vitripennis* lines (Kruskal-Wallis: H=115.99, p < 0.001).

*w*A*w*B(PU) had shorter average life span compared to the two single infected lines {MWU, U = 6130, p < 0.0001 for *w*A(PU) and U = 6604, p < 0.0001 for *w*B(PU)}. 0(PU) showed significantly longer average life span compared to all the other infected lines {MWU, U = 2324, p < 0.05 for *w*A(PU); U = 6261, p < 0.0001 for *w*B(PU), and 8276.5, p < 0.0001 for *w*A*w*B(PU)}.

The recently cured lines 0(*w*A PU), 0(*w*B PU) and 0(*w*A*w*B PU) showed significantly longer average life span than their parental lines *w*A(PU) {MWU: U = 1957.5, p < 0.01}, *w*B(PU) (MWU: U = 2324.5, p < 0.05) and *w*A*w*B(PU) {MWU: U = 441.5, p < 0.0001} respectively, and were comparable to the uninfected line 0(PU) {MWU: U = 3256, p = 0.36 for *w*A(PU), MWU: U = 3484, p = 0.25 and MWU: U = 1808, p = 0.66 for *w*A*w*B(PU)}.

1. A significant difference was observed between the average life span of the females of different *N. vitripennis* lines (Kruskal-Wallis: H=298.09, p < 0.001).

The single A infected *w*A(PU) females, showed the shortest average life span {MWU: U = 3112.5, p < 0.0001 for *w*A*w*B(PU), U = 1331, p < 0.0001 for *w*B(PU) and U = 5307, p < 0.0001, for 0(PU)} followed by *w*A*w*B(PU) { MWU: U = 6500, p < 0.0001 for *w*B(PU) and U = 4452.5, p < 0.0001 for 0(PU)}. 0(PU) and *w*B(PU) females showed similar life spans { MWU: U = 3027, p = 0.24}.

The recently cured lines of 0(*w*A PU), 0(*w*B PU) and 0(*w*A*w*B PU) showed significant increase in the average life span when compared to their parent lines *w*A(PU) {MWU: U = 96, p < 0.0001}, *w*B(PU) {MWU: U = 1746.5, p < 0.0001} and *w*A*w*B(PU) {MWU: U = 1608, p < 0.0001} respectively. The recently cured lines showed longer average life span than 0(PU) {MWU: U = 1012.5, p < 0.0001 for 0(*w*A PU), U = 1667, p < 0.0001 for 0(*w*B PU), U = 3112.5, p < 2370.5 for 0(*w*A*w*B PU)}.


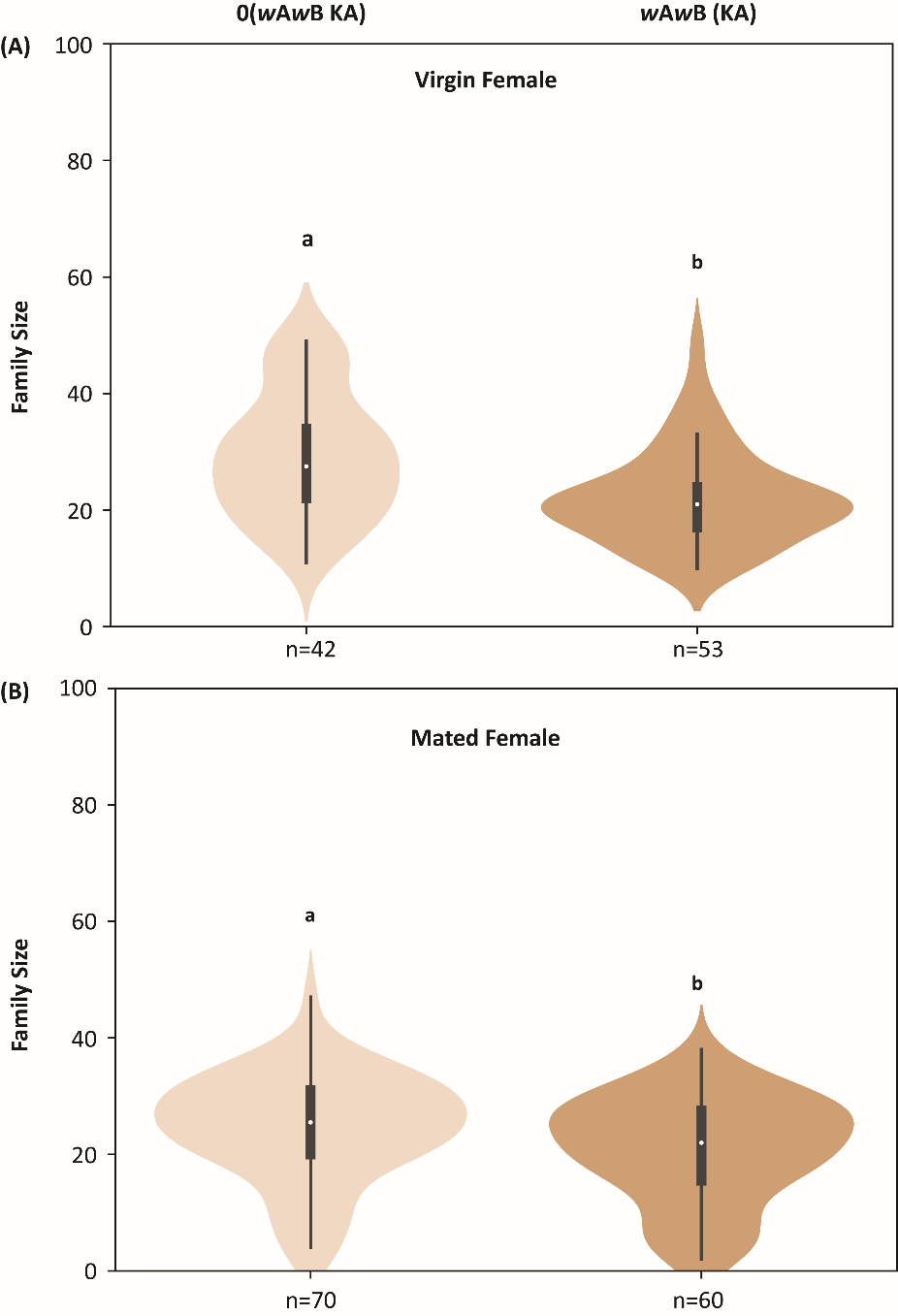


**Figure S3. Progeny family size of *N. vitripennis* line NV-KA.**

Family size produced by females when hosted as virgins (A) and mated (B). The statistical significance is tested using the Mann-Whitney U test with p < 0.05.
